# Extranodal follicular dendritic cell sarcoma: A clinicopathological report of four cases and a literature review

**DOI:** 10.3892/ol.2014.2681

**Published:** 2014-11-07

**Authors:** RUI-FEN WANG, WEI HAN, LEI QI, LI-HUI SHAN, ZHENG-CAI WANG, LI-FENG WANG

**Affiliations:** 1 Department of Pathology, Xin Hua Hospital Affiliated to Shanghai Jiaotong University School of Medicine, Shanghai 200092, P.R. China; 2 Department of Pathology, The First Hospital of Harbin Medical University, Harbin 150001, P.R. China; 3 Department of Pathology, The Fourth Hospital of Harbin Medical University, Harbin 150001, P.R. China

**Keywords:** follicular dendritic cell sarcoma, extranodal, literature review

## Abstract

The aim of the present study was to characterize the clinicopathological features of follicular dendritic cell sarcoma (FDCS), and to report the experience of the Xin Hua Hospital Affiliated to Shanghai Jiaotong University School of Medicine (Shanghai, China) with this entity. The clinicopathological findings of four cases that had recently been encountered and 142 previously reported cases in the English literature were evaluated. The current tumors were found in two male and two female patients, aged 49–76 years old, who exhibited a mean tumor size of 8.7 cm. Three of the four cases were misdiagnosed during the initial diagnosis and one experienced intra-abdominal recurrence six months after the first diagnosis. Assessment of all 142 cases in the literature revealed a mild female predominance. The tumors exhibited a mean tumor size of ~7.0 cm. Histologically, the tumors were composed of plump spindle- or oval-shaped cells that exhibited eosinophilic cytoplasm and were arranged in sheets, storiform patterns or whorls. Immunohistochemically, the neoplastic cells expressed at least one of the FDC markers. Among the 130 cases with follow-up data, the overall recurrence, metastasis and mortality rates were 49.2% (64 cases), 21.5% (28 cases), and 13.8% (18 cases), respectively. FDCS can appear deceptively similar to other soft-tissue tumors, even poorly-differentiated carcinomas. A correct diagnosis requires a high degree of suspicion and immunohistochemical evaluation.

## Introduction

Follicular dendritic cells (FDCs), which exhibit a dendritic configuration and occasional multinucleation, can be found in primary and secondary lymphoid follicles at nodal or extranodal sites. FDCs are characterized by indistinct borders, oval nuclei, delicate nuclear membranes, faintly eosinophilic cytoplasm, small but distinct nucleoli, and their reaction to cluster of differentiation (CD)21, CD23, CD35, fascin and clusterin (1). The cells play a major role in antigen presentation and antigen-dependent maturation of the B-cell immune response (2). Based on the normal distribution of FDCs, FDC sarcoma (FDCS) presents within the lymph nodes and at extranodal sites. Since FDCS has been fairly well-characterized morphologically and possesses a distinct immunophenotype, the possibility of diagnosis is currently more readily established for spindle cell lesions in the lymph nodes. However, extranodal FDCS is less well recognized, although its occurrence has been noted since 1994 (3). In the present study, the literature was reviewed in order to obtain a deeper recognition of FDCS arising from extranodal sites. Prior to 2013, there were only 142 cases of extranodal FDCS reported in the English language literature, with up to one-fourth of those cases being initially misdiagnosed. In the current study, four cases of extranodal FDCS are presented, and the clinicopathological features of extranodal FDCS reported in the literature are reviewed.

## Materials and methods

### Samples

The four cases were retrieved following consultation at the Xin Hua Hospital Affiliated to Shanghai Jiaotong University School of Medicine (Shanghai, China). One case has already been reported as FDCS (4), but additional tests have since been performed. The clinical information was obtained from the hospital and outpatient records, and from the contributing pathologists and clinicians. Histological materials were processed in a routine manner, with formalin fixation and paraffin embedding for hematoxylin and eosin staining. Ethical approval for this study was obtained from the Ethics Committee of Xin Hua Hospital Affiliated to Shanghai Jiaotong University School of Medicine.

### Immunohistochemistry

Immunohistochemical staining was performed on the formalin-fixed, paraffin-embedded tissue sections using the EnVision method (Leica BOND-MAX, Leica Biosystems, Newcastle Upon Tyne, UK). The antibodies used in the immunohistochemistry were for CD21, CD23, CD35, vimentin, CD20, CD3, desmin, CD68, cytokeratin (CK), CD117, CD34, thyroid transcription factor-1, S100 protein, human melanoma black 45 (HMB45), smooth muscle actin (SMA), epithelial membrane antigen (EMA) and Ki-67. Appropriate positive and negative controls were simultaneously evaluated.

### Literature review

Previous studies were obtained from the MEDLINE database, a major index literature source, using the term ‘follicular dendritic cell sarcoma/tumor’. Nodal FDCS was not included. An effort was made to identify cases that had been reported more than once and only the case with the most recently updated information was included in the present report. A total of 142 cases of extranodal FDCS were retrieved from the English literature (5–86). A total of 11 cases were excluded from the analysis due to omitted clinical data.

### Statistical analysis

The association between the various clinicopathological features and a higher event rate (recurrence, metastases, and mortality) were assessed using the χ^2^ test. Data analyses were generated using SPSS for Windows, version 13.0 (SPSS, Inc., Chicago, IL, USA). P<0.05 was used to indicate a statistically significant difference.

## Results

### Report of four cases

#### Clinical data

The case summaries of the present four cases of extranodal FDCS are shown in Table I. The tumors were located in the tonsils, stomach, liver and lungs, respectively.

#### Histopathological findings

**Figure 1:**
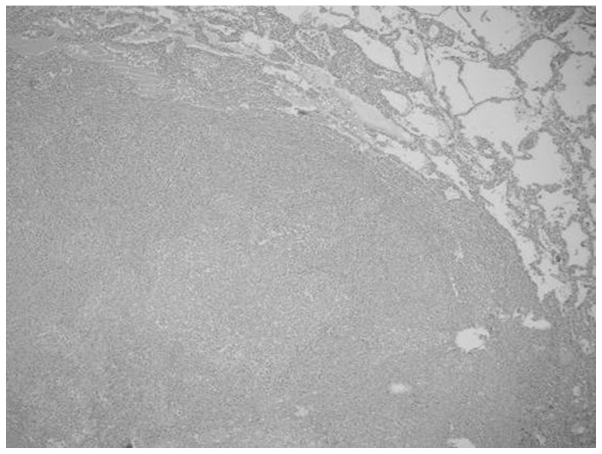
Case four: Follicular dendritic cell sarcoma in the lung exhibiting sharp demarcation from the residual parenchyma.

**Figure 2:**
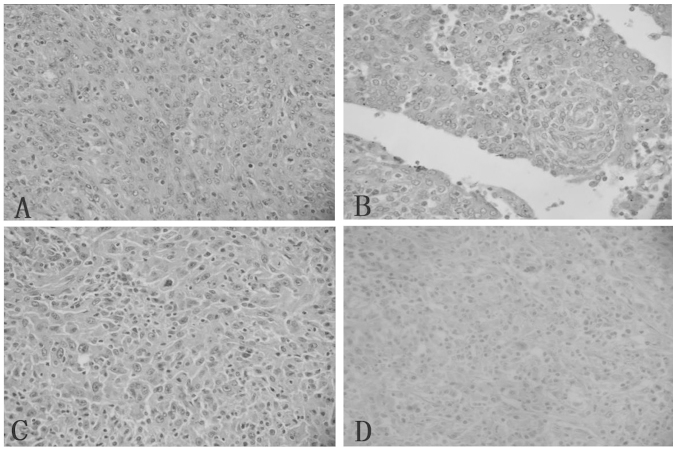
Growth patterns of follicular dendritic cell sarcoma demonstrating the characteristic sprinkling of small lymphocytes throughout the tumor. (A) Case two: Interlacing fascicles of spindled cells with oval nuclei, empty nucleoplasm and distinct small nucleoli. (B) Case one: Whorled pattern mimicking a feature of meningioma. (C) Case four: Diffuse growth pattern with multinucleated tumor cells. (D) Case three: Storiform pattern with intranuclear pseudoinclusion.

Macroscopically, the four tumors were well-circumscribed, exhibiting a solid, nodular, grey-yellow or dust-colored cut surface, similar to sarcoma or lymphoma. Microscopically, the tumors were relatively well-circumscribed (Fig. 1). Cases one and four revealed an epithelioid cell morphology, with a whorled, diffuse or trabecular growth pattern, while cases two and three exhibited an increased proportion of spindled cells. The growth pattern in these cases was fascicular, storiform and whorled (Fig. 2). In all cases, the tumor cells possessed a moderate amount of faintly eosinophilic cytoplasm and indistinct cell borders. Binucleated or multinucleated tumor cells were occasionally observed. The nuclei were oval to elongated in shape, with thin and lightly-stained purplish nuclear membranes, vesicular or stippled chromatin and distinct nucleoli. Certain nuclei contained round intranuclear pseudoinclusions (Fig. 2). Mitoses were not prominent and were absent in three cases and in 6–8/10 high-power fields (HPFs) in case two. Necrosis was observed in three cases. There were small lymphocytes scattered throughout the tumors and clustered around vessels. In case two, certain irregular pseudolacuna-like blood vessels were found, the insides of which exhibited plump eosinophilic protein-like liquids that resembled the perivascular spaces observed in thymoma.

#### Immunohistochemical findings

**Figure 3:**
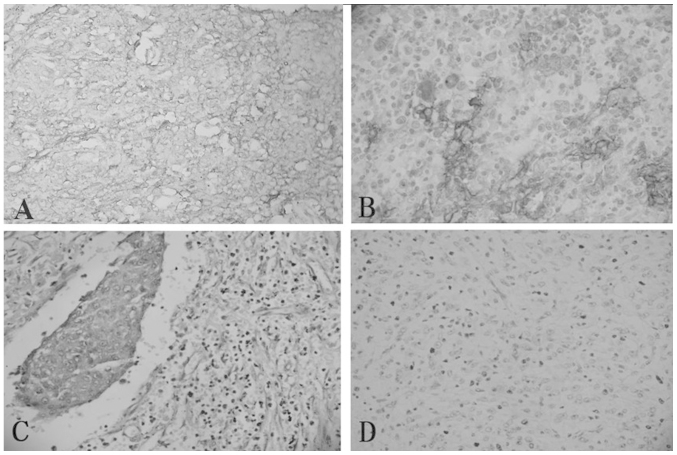
Immunohistochemical staining. (A) Case one: Positive staining for cluster of differentiation (CD)21. (B) Case four: Positive staining for CD23 demonstrating a trabecular growth pattern. (C) Case one: Focal staining for EMA. (D) Case two: A Ki-67 index of 15%.

The staining of CD21, CD23, CD35 and vimentin was performed for all four cases, while the staining of CD68, S-100 and EMA was performed for only three cases. Immunohistochemically, all four cases were diffusely positive for CD21, CD23 and vimentin expression, and focally positive for CD35 expression. Two of three cases were focally positive for CD68 expression, and one of three cases was focally positive for S-100 and EMA expression (Fig. 3). Case two exhibited a negative result for the expression of GIST markers CD34 and CD117, while case four exhibited a negative result for thyroid transcription factor and the melanoma marker HMB45. Staining for these markers was not performed in the other cases. All the present cases were negative for CK and SMA. For the associated lymphocytes, T cells (CD3^+^) outnumbered B cells (CD20^+^). The Ki-67 labeling index of case one was >1%, while the indexes of cases two and four were 15 and 40%, respectively.

### Literature review

#### Clinical features

A total of 142 cases of extranodal FDCS were retrieved from the literature. The patients, 77 females and 65 males (female to male ratio, 1.2:1), ranged in age from 9–82 years old. The tumor affected various anatomical sites. The more predominant extranodal sites of tumor involvement were the tonsils (28), pharynx (19), liver (18), spleen (17), intra-abdominal but external viscera (14), soft tissue of the neck (11), mediastinum (6), lungs (4), gastrointestinal tract (three in the stomach, four in the small bowel, two in the large intestine and one in the anal canal), thyroid, breast, palate, parotid gland (3) and pancreas (2). Other organ sites consisted of the intracalvarium, pleura, dura mate spinalis, pelvis and soft tissue of the thigh, with one case occurring in each location. In total, 12 cases exhibited lymph node and extranodal FDCS, and 9 cases exhibited distant metastases at the time of presentation.

#### Pathological findings

The average size of the tumors in all sites was 7.0 cm, with a range of 1–22 cm. Intra-abdominal tumors (n=55) exhibited an average tumor size of 10.2 cm (range, 3–22 cm), whereas the average tumor size of extra-abdominal sites (n=72) was only 5.4 cm (range, 1–16 cm) (P<0.05). Data regarding tumor size were unavailable for 15 cases.

The macroscopic and microscopic findings of the cases from the literature were similar to those described in the present four cases. Macroscopically, the tumors were well-circumscribed with a solid, nodular, grey-yellow or dust-colored cut surface, similar to sarcoma or lymphoma. Microscopically, the tumors were circumscribed from the surrounding parenchyma, with or without a fibrous capsule. The tumor cells varied from plump and spindled to epithelioid, with a moderate amount of faintly eosinophilic cytoplasm and indistinct cell borders. Binucleated or multinucleated tumor cells were occasionally observed. The nuclei were oval to elongated in shape, with a thin and lightly stained purplish nuclear membrane, vesicular or stippled chromatin, and distinct nucleoli. Intranuclear pseudoinclusions were found in certain cases. A mitotic count was available in 80 cases, with a median of 4/10 HPFs ranging between 0 and 50/10 HPFs. In total, 51 cases possessed a mitotic count of <5/10 HPFs. Information on necrosis was available in 70 cases, of which 34 cases (48.6%) exhibited coagulative necrosis. There were small lymphocytes scattered throughout the tumors and clustered around vessels.

Immunohistochemically, all the cases were positive for at least one of the FDC markers, including CD21, CD23, CD35, fascin and clusterin, and were negative for CK. Staining for CD117, CD34, desmin, HMB45 and CD1α, CD68, S100, and EMA revealed variable results among the different cases. Desmoplakin, a desmosome-associated protein, was detected in the majority of the cases. The associated lymphocytes were more commonly T cells (CD3^+^) rather than B cells (CD20^+^). Prominent cell processes that were focally joined by well-formed cell junctions and desmosome-like cytoplasm were exhibited ultrastructurally by the tumor cells.

#### Discordant diagnoses at the initial evaluation

Overall, 40 cases were misdiagnosed at the time of the initial evaluation. In each of these cases, the diagnosis of FDCS was not considered at the initial evaluation. The disease entities that were considered consisted of interdigitating reticulum cell sarcoma, minor salivary gland tumors, malignant peripheral nerve sheath tumors, reactive response, inflammatory pseudotumors, malignant fibrous histocytomas, meningioma, mesenchymal tumors with neural differentiation, schwannoma, Hodgkin’s lymphoma, angiosarcoma, malignant melanoma, large-cell lymphoma, spindle cell carcinoma, primitive neuroectodermal tumors, malignant myoepithelial carcinoma and gastrointestinal stromal tumors.

#### Correlation between clinicopathological findings and patient outcome

Follow-up information was available in 130 cases, with a follow-up duration period of 0.5 to 324 months (mean, 34.5 months). The overall recurrence, metastasis and mortality rates were 49.2% (64 cases), 21.5% (28 cases), and 13.8% (18 cases), respectively. Local recurrence occurred in 25 patients (20.5%) following resection (range, 6–180 months). Distant metastases occurred in 28 patients and the metastatic sites included the liver, lung, bones, ovaries, thyroid gland, omentum, lymph nodes and soft tissue. At follow-up, it was determined that 18 (13.8%) patients had succumbed to the disease, 38 (29.2%) were alive with the disease, and 74 (56.9%) patients were alive with no evidence of the disease. Both clinicopathological features and follow-up information were available for 109 patients; the association between the various clinicopathological features of early-stage disease and a higher event rate (recurrence and mortality) was analyzed for these patients, the results of which are summarized in Table II. A large tumor size (≥7.5 cm) and high mitotic rate (≥5/10 HPF) were associated with mortality, but not recurrence.

## Discussion

The majority of FDCS occurs in adults, with a mild female predilection. The clinical characteristics of FDCS include a painless mass. It is important that doctors are aware of FDCS and are able to recognize this tumor, as it is extremely similar to a wide range of other tumors and tumor-like lesions. Usually, FDCS is not considered in routine diagnoses, particularly when it occurs in extranodal sites. FDCS can be missed even after immunohistochemical studies, as the markers for FDC are not included among the routine antibody panel that is used for the investigation of poorly-differentiated neoplasms. Thus, tumors arising from extranodal sites are often misdiagnosed or excluded during the initial diagnosis, and this is corrected only when there is tumor recurrence or metastasis (78). In the present four cases, three were misdiagnosed, which could lead to unnecessary treatment and associated morbidity. This prompted the investigation of potential diagnostic pitfalls and a detailed analysis of extranodal FDCS.

The diagnosis of FDCS is established based on morphological and immunohistochemical findings. Histologically, the tumors in the present study were composed of spindle to oval-shaped cells, which were arranged in sheets, interlacing fascicles or whorls. The tumor cells exhibited plump eosinophilic cytoplasm, with ill-defined cell borders, forming a less diffuse growth pattern. The nuclei of the tumor cells were small, oval or round in shape, and exhibited a vesicular chromatin pattern. Certain multinucleate tumor cells were also present. Nuclear pleomorphisms and scattered mitotic figures, often <3 mitoses/10 HPFs, were observed. The neoplastic cells were intermixed with small mature lymphocytes and plasma cells, which surrounded the blood vessel and formed a cuff-like structure.

The correct diagnosis of FDCS must also be made from immunohistochemistry studies. Ultrastructural studies are desirable when making a diagnosis of FDCS, but are not essential. FDCS cells demonstrate a similar immunophenotype to normal FDCs. CD21, CD23, CD35, fascin, and clusterin are more specific markers for FDCS. CD68, S-100, EMA, and LCA are variably immunoreactive, while results for CK, CD1α, CD31, CD34, and HMB-45 are negative (84). A previous study demonstrated that EGFR and clusterin exhibit high specificity for diagnosis (1). Fascin has been revealed to be extremely non-specific among spindle cell tumors, indicating that they do not have a follicular dendritic lineage (1). High D2–40 expression has been revealed in FDCS, while weak or no expression has been found in other dendritic cell tumors (41,87). Electron microscopy reveals that the tumor cells have long, slender cytoplasmic processes, which were connected by desmosomes, few cellular organs and no Birbeck granules (78).

Although FDCS possesses certain morphological and immunophenotypical features, those that occur in extranodal sites are particularly rare and extremely similar to other soft-tissue tumors, even poorly-differentiated cancers. For example, in the cases from the literature review, FDCS of the parapharyngeal region was misdiagnosed as ectopic meningioma (24), pars palatalis was misdiagnosed as acinic cell carcinoma (88) and primary FDCS of the alimentary tract was misdiagnosed as mesenchymal neoplasm of the abdominal cavity (43), diffuse large-cell lymphoma of the liver (8) and low-degree malignant tumor of the mesentery (57).

Therefore, an awareness of the morphological features of FDCS and the appropriate application of FDC markers for any tumors exhibiting an unusual appearance, should aid a correct diagnosis (74). In case two of the present study, stomach FDCS was misdiagnosed as a GIST, in which the tumor cells usually exhibit less eosinophilic cytoplasm compared with the cells of spindle cell tumors, which exhibit greater specific differentiation, similar to smooth muscle and neurogenic tumors. Immunohistochemically, the tumor cells of GISTs are positive for CD117 and CD34, but negative for FDC markers. Another type of neoplasm that has a dendritic cell origin is interdigitating dendritic cell sarcoma, which also shares certain immunophenotypical, histological and ultrastructural features with FDCS. However, interdigitating dendritic cell sarcoma is S-100-positive, but negative for FDC markers. When FDC markers are present and there is no CD45 expression, the diagnosis of interdigitating dendritic cell sarcoma can be excluded. Sparse intracytoplasmic organelles and interdigitating cytoplasmic processes are typical ultrastructural features, but interdigitating dendritic cell sarcoma does not exhibit desmosomes. In addition, a differential diagnosis should include malignant melanoma, ectopic meningioma, malignant fibrohistiocytoma, ectopic thymoma, malignant peripheral nerve sheath tumor, lymphepithelioma and malignant lymphoma. Among these possibilities, malignant melanoma is positive for HMB-45 and ectopic meningioma is positive for vimentin and EMA. Malignant fibrohistiocytoma is characterized by spindle cells in a storiform pattern, multinucleate tumor giant cells, marked cytological atypia and positive histiocyte marker expression. Ectopic thymoma is positive for CK and terminal deoxynucleotidyl transferase expression in lymphocytes. However, all the aforementioned lesions are negative for FDC markers (77).

The etiopathogenesis of FDCS remains unclear. Novel investigations have revealed that FDCS was associated with hyaline-vascular Castleman’s disease, in which FDC hyperplasia can develop into FDCS, similar to certain nodal counterparts (20). Cheuk *et al* (45) reported a series of inflammatory pseudotumor (IPT)-like FDCS, which were positive for Epstein-Barr virus-encoded RNA (EBER) in all cases. Shia *et al* (14) suggested that the IPT-like FDCS may represent an earlier stage of FDCS, which may exert an inciting factor associated with EBV. Upon review of studies of cases that had previously been tested for EBV, it was discovered that 10 out of 11 cases of hepatic FDCS exhibited positive EBER expression by *in situ* hybridization (47), whereas 11 out of 17 splenic cases were positive for the virus. EBV was not detected in any of the cases located in other sites. Recently, it was found that tumor cells were uniformly positive for EBV in all six studied cases of IPT-like FDC sarcoma of the spleen, and there were predominant immunoglobulin (Ig)G4^+^ plasma cells in the tumor nodules, suggesting that IPT-like FDC sarcoma of the spleen may be closely associated with EBV and the IgG4-associated disease (86). Therefore, there is a pathogenetic difference between liver and splenic FDCS and other FDCS. However, EBV was not detected in case three, an FDCS of the liver, in the present study. In addition, certain studies revealed that FDCS coexisting with myasthenia gravis was also associated with immature T cells. These cases suggested that myasthenia gravis may be a paraneoplastic manifestation of FDCS and that FDCS may be capable of mediating aberrant immune activation (89–91). Other data suggested that the p53-mediated pathway may possibly be involved in the development or progression of FDCS (75). However, the association was not evident in any lesion in the present cases.

The biological behavior of FDCS is difficult to predict, as it is generally considered to be a low-grade soft-tissue sarcoma. Although originally known as an FDC tumor, the term FDCS was proposed by Chan *et al* (6) in order to emphasize the clinical behavior of the neoplasm as a sarcoma rather than a lymphoma. Until recently, the tumor was believed to be indolent with a tendency to recur, but with a low risk of metastasis. Studies of larger cohorts and for longer follow-up periods have since concluded that FDCS is more aggressive than previously hypothesized, and that the tumor should be considered as an intermediate-grade malignancy at least (6,92,93). In the present study, a large tumor size and a high mitotic rate were found to be associated with mortality, but an intra-abdominal location and the presence of necrosis were not associated with a poor prognosis. However, other studies have found that poor prognostic factors included an intra-abdominal location, a tumor size of >6 cm, a high mitotic rate, necrosis and nuclear pleomorphism (92,93,14). In case two of the present study, cell atypia and 6–8 mitoses/10 HPFs were observed, which indicated a poor prognosis. There were multiple metastases in the liver at the time of surgery. In addition, the marked intratumoral immature T-cell burden may also perform a role as an adverse prognostic factor in FDCS (89). Radical resection of the tumor is the primary therapy, but the value of radiotherapy and chemotherapy in the treatment of this neoplasm remains uncertain.

In conclusion, extranodal FDCS is a rare, often misdiagnosed, malignant tumor. An increased awareness of the morphological spectrum of FDCS and appropriate immunostains for FDC differentiation should aid in the recognition of FDCS. It is imperative for the biological behavior of this tumor to be clearly recognized. Increased awareness of the existence of FDCS may aid a reduction in the potential for diagnostic error.

## Figures and Tables

**Table I:** Clinical data of four novel cases of extranodal follicular dendritic cell sarcoma.

Case	Gender	Age	Site	Maximal size, cm	Manifestation	Initial diagnosis	Treatment	Outcome at follow-up (months)
1	M	65	Tonsil	No data	Tonsil pain	SCC	Tonsillectomy	NED (25)
2	F	53	Stomach	14	Debilitation, abdominal distention, anepithymia	GIST	Surgery Radiotherapy Hemotherapy	AWD (10)
3	M	49	Liver	9	Right upper quadrant pain	Inflammatory pseudotumor	Surgery	Intra-abdominal recurrence (8)
4	F	76	Lung	3	Dyspnea	Malignant melanoma	Surgery	Succumbed to disease

NED, no evidence of disease; AWD, alive with disease; SCC, squamous cell carcinoma; GIST, gastrointestinal stromal tumor.

**Table II:** Analysis of factors associated with a higher risk of recurrence or mortality in localized disease.

Parameter	Disease-free, n	Recurrence, n	Mortality, n	P-value^a^	P-value^b^
Age, years				0.017	0.752
≥50	38	10	7		
<50	25	21	8		
Gender				0.310	0.765
Female	34	20	8		
Male	29	11	7		
Tumor size, cm				0.391	0.006
≥7.5	18	7	9		
<7.5	41	18	4		
Location				0.128	0.095
Intra-abdominal	28	7	9		
Extra-abdominal	36	23	6		
Mitotic count				0.304	0.004
≥5/10 HPFs	7	8	6		
<5/10 HPFs	31	11	1		
Necrosis				0.012	0.367
Present	24	5	4		
Absent	14	12	1		

a Factors associated with recurrence;

b factors associated with mortality.

HPFs, high-power fields.
